# Sequential almonertinib therapy following osimertinib-induced interstitial lung disease in early-stage EGFR-mutant lung adenocarcinoma: a case report

**DOI:** 10.3389/fonc.2026.1699804

**Published:** 2026-05-08

**Authors:** Jianbin Zhang, Sishi Huang, Hui Shen

**Affiliations:** 1 Department of Thoracic Surgery, Huzhou Central Hospital, Affiliated Central Hospital of Huzhou University, Huzhou, Zhejiang, China; 2 Department of Respiratory Medicine, Huzhou Central Hospital, Fifth School of Clinical Medicine of Zhejiang Chinese Medical University, Huzhou, Zhejiang, China

**Keywords:** adjuvant therapy, almonertinib, case report, EGFR-TKI, interstitial lung disease, non-small cell lung cancer, osimertinib

## Abstract

**Background:**

Osimertinib is the standard adjuvant therapy for resected epidermal growth factor receptor (EGFR)-mutant non-small cell lung cancer (NSCLC). However, its use is limited by osimertinib-induced interstitial lung disease (Osi-ILD).

**Case Presentation:**

We describe the case of a 62-year-old woman with resected stage IIB (pT1cN1M0) EGFR L858R-mutant lung adenocarcinoma who developed grade 2 Osi-ILD, characterized by new bilateral ground-glass opacities on high-resolution computed tomography (HRCT), after 3 months of adjuvant osimertinib therapy. Osimertinib was immediately discontinued, leading to complete symptomatic and radiographic resolution within 8 weeks without corticosteroid therapy. Given the high risk of ILD recurrence upon rechallenge and the suboptimal efficacy of chemotherapy, a multidisciplinary team consensus recommended switching to almonertinib (110 mg once daily). Remarkably, the patient has maintained disease-free survival without ILD relapse for 36 months.

**Conclusion:**

This case provides clinical evidence that switching to almonertinib may represent a viable and safe therapeutic strategy, thereby addressing a critical unmet need in the management of resected early-stage EGFR-mutant NSCLC after Osi-ILD.

## Introduction

Osimertinib, a third-generation epidermal growth factor receptor tyrosine kinase inhibitor (EGFR-TKI), is the standard adjuvant therapy for patients with resected stage IB-IIIA EGFR-mutant non-small cell lung cancer (NSCLC) (1). However, its clinical application is significantly constrained by interstitial lung disease (ILD), a potentially fatal toxicity observed in 2–4% of patients in the pivotal ADAURA trial (2) and potentially at higher incidence rates in real-world populations (3). Osimertinib-induced ILD (Osi-ILD) forces permanent osimertinib discontinuation, consequently elevating the risk of tumor recurrence.

Optimal management strategies following Osi-ILD are not well established. While adjuvant chemotherapy represents the guideline-recommended alternative, it is associated with inferior disease-free survival (DFS) and overall survival (OS) outcomes compared to EGFR-TKIs in patients with EGFR-mutant disease (4). Rechallenging with osimertinib is associated with an unacceptably high risk of ILD relapse (5). This therapeutic dilemma necessitates the exploration of alternative strategies. Almonertinib, a structurally distinct third-generation EGFR-TKI (6), has emerged as a promising candidate based on its demonstrated favorable pulmonary safety profile in patients with metastatic NSCLC following Osi-ILD (7). However, its role in the curative-intent adjuvant setting remains unexplored. More critically, the management of patients who develop Osi-ILD during adjuvant therapy presents a profound and urgent clinical challenge: permanent discontinuation of osimertinib forfeits its unmatched efficacy, rechallenge is contraindicated, and adjuvant chemotherapy yields suboptimal disease control.

Herein, we report the first documented case of successful sequential therapy with full-dose almonertinib (110 mg once daily) in the adjuvant setting for early-stage EGFR-mutant lung adenocarcinoma following Osi-ILD, which has maintained disease-free survival without ILD relapse for 36 months.

## Case presentation

**Figure 1:**
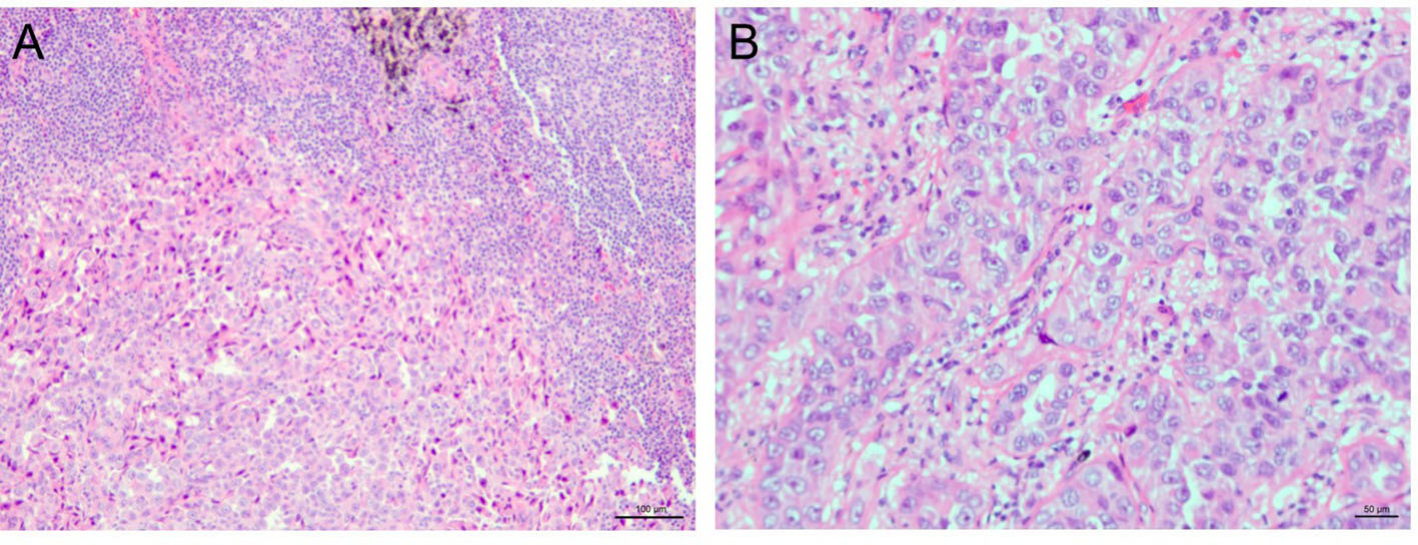
Histopathological findings of the resected tumor (pT1cN1M0, stage IIB). **(A)** Hematoxylin and eosin (H&E) staining of the primary lung tumor (20x magnification) shows an acinar-predominant invasive lung adenocarcinoma with solid components (15%). **(B)** H&E staining of a metastatic lymph node (station 12R) (10x magnification) confirms nodal involvement.

In November 2022, a 62-year-old never-smoked woman with no history of pulmonary disease underwent a right upper lobectomy and systematic lymph node dissection in our department. The final pathological examination confirmed an acinar-predominant invasive lung adenocarcinoma with a solid component (15%) and metastasis in a station 12 lymph node, definitively staged as pT1cN1M0 (stage IIB, according to the 8th edition of the AJCC staging system) (**Figure 1**). Molecular profiling via amplification refractory mutation system polymerase chain reaction (ARMS-PCR) identified an EGFR exon 21 L858R mutation. Testing for ALK, ROS1, and other driver alterations was negative.

**Figure 2:**
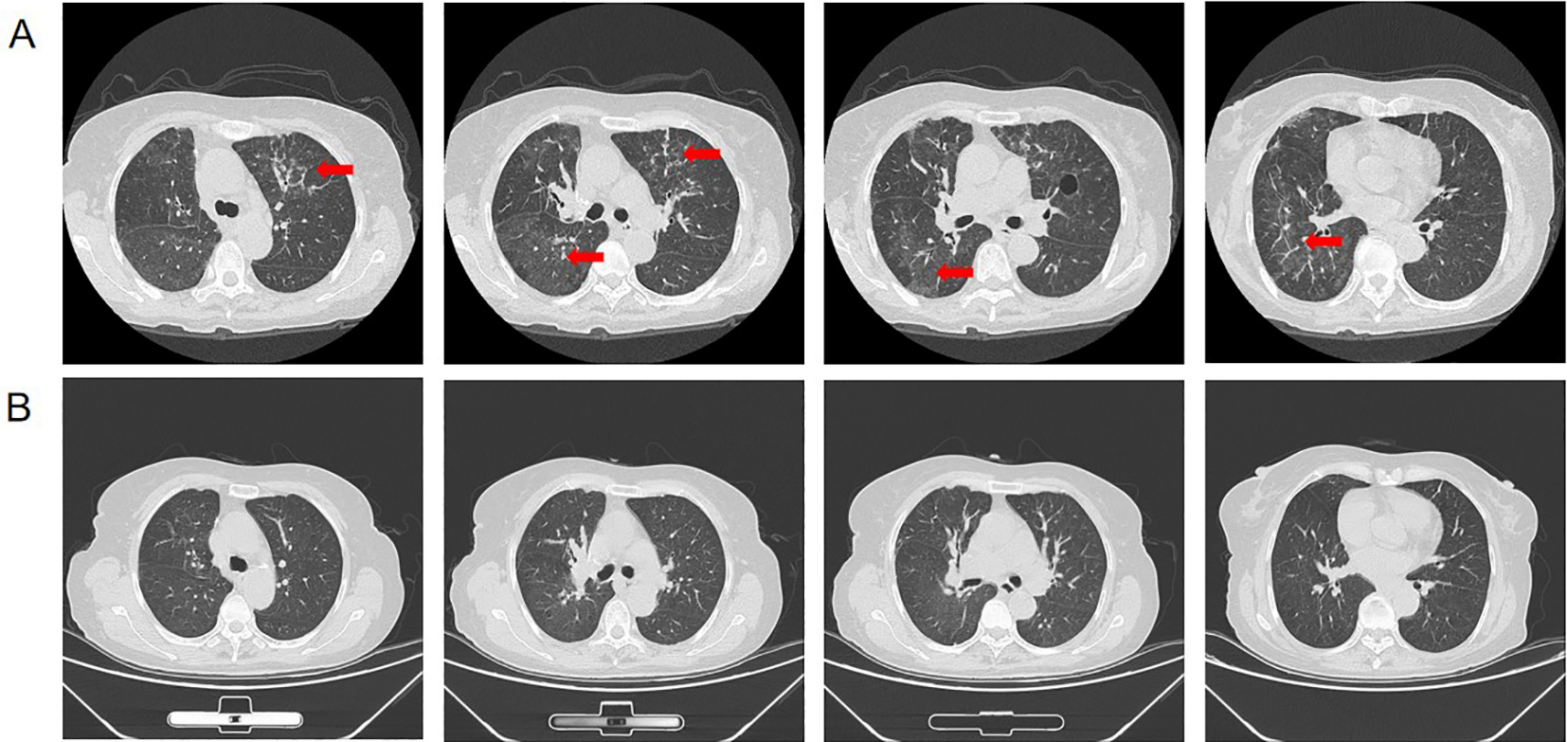
Serial thoracic HRCT before and after management of Osi-ILD. **(A)** Thoracic HRCT obtained at the onset of symptoms after 3 months of osimertinib therapy shows characteristic bilateral, confluent GGOs with fine reticulation, consistent with Osi-ILD. **(B)** Follow-up HRCT acquired 8 weeks after osimertinib discontinuation confirms complete radiological resolution of the interstitial lung abnormalities.

Adjuvant osimertinib (80 mg once daily) was initiated in January 2023. Three months later, the patient developed progressive dyspnea and chest tightness without infectious symptoms. Examination revealed bibasilar inspiratory crackles. Laboratory findings excluded infection (WBC 6.8×10^9^/L, CRP 5 mg/L, PCT <0.05 ng/mL). Serum BNP was 35.4 pg/mL (normal is <100 pg/mL), and cardiac enzymes and troponin I were within normal limits. An electrocardiogram showed a normal sinus rhythm without ischemic changes or arrhythmias. A chest high-resolution computed tomography (HRCT) scan performed with a 1.25 mm slice thickness revealed new bilateral diffuse ground-glass opacities (GGOs) and fine reticulation, predominantly in the peripheral and basal regions (**Figure 2A**). Following a multidisciplinary team (MDT) consultation, a diagnosis of Osi-ILD, graded as Common Terminology Criteria for Adverse Events (CTCAE) Version 5.0 Grade 2 (8), was established based on the temporal association with osimertinib initiation, characteristic HRCT findings, and exclusion of competing etiologies. Osimertinib treatment was immediately discontinued. Given the risk of progression, stringent monitoring was instituted, comprising frequent clinical assessments and educating the patient on recognizing symptom deterioration. The patient’s symptoms spontaneously resolved following drug discontinuation without any corticosteroid intervention. Complete resolution of the bilateral pulmonary lesions was confirmed by follow-up HRCT performed 8 weeks later (**Figure 2B**).

**Figure 3:**
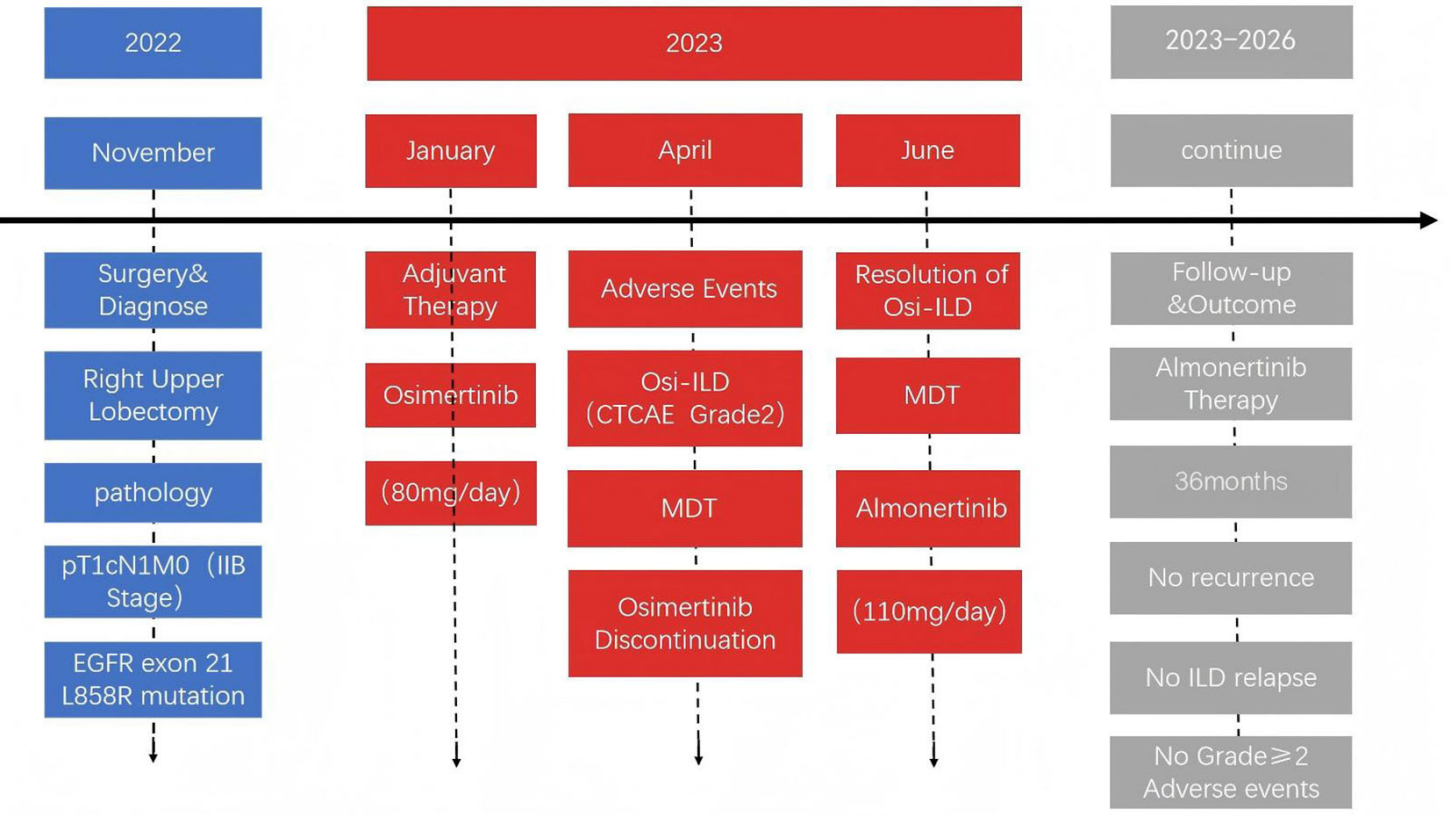
Timeline of key clinical events. Blue: Surgical diagnosis (Nov 2022) of stage IIB EGFR L858R-mutant lung adenocarcinoma. Red: Adjuvant osimertinib (Jan–Apr 2023), discontinued due to grade 2 Osi-ILD, with a subsequent switch to almonertinib. Gray: Almonertinib therapy (Jun 2023–Jan 2026), with the patient maintaining event-free survival throughout the entire 36-month postoperative follow-up period, without recurrence, ILD relapse, or significant toxicity.

Nevertheless, the high risk of recurrence associated with stage IIB disease necessitated ongoing adjuvant therapy, presenting a significant clinical challenge. Consequently, a repeat MDT consultation was convened, and a consensus-based, risk-adapted therapeutic strategy was formulated. Given the suboptimal efficacy of platinum-based chemotherapy in treating EGFR-mutant NSCLC and the high risk of ILD recurrence upon osimertinib rechallenge, the MDT unanimously advocated for a switch to almonertinib treatment. Following comprehensive risk-benefit deliberation and documented informed consent, the patient initiated almonertinib therapy at 110 mg once daily in June 2023. She was subsequently enrolled in a structured surveillance protocol, comprising monthly clinical assessments and quarterly imaging surveillance. Through January 2026 (36 months of follow-up), the patient’s safety profile remained favorable, with no recurrent ILD or other treatment-emergent adverse events (TEAEs). Serial surveillance imaging confirmed the absence of locoregional recurrence or distant metastasis. The treatment timeline and chronology of key clinical events are summarized in **Figure 3**.

## Discussion

In this case, the patient developed CTCAE grade 2 Osi-ILD 3 months after osimertinib initiation, which resolved completely within 8 weeks of discontinuing the drug without corticosteroid intervention. Given the high recurrence risk associated with stage IIB NSCLC and the high risk of recurrent ILD upon osimertinib rechallenge, almonertinib therapy was initiated following a comprehensive MDT consensus. Notably, at the 36-month follow-up, the patient had maintained durable disease control without recurrent ILD or significant almonertinib-related toxicities. To the best of our knowledge, this represents the first successful application of sequential almonertinib specifically in the adjuvant setting following Osi-ILD. While TKI switching due to toxicity has been reported in advanced NSCLC, the adjuvant setting presents a uniquely high-stakes context in which therapeutic failure equates to a lost opportunity for a cure.

The markedly lower incidence of ILD with almonertinib compared to osimertinib offers a compelling mechanistic rationale for the success of this sequential strategy. While osimertinib is associated with a 2–4% risk of ILD in the adjuvant setting (2) and a >30% risk of recurrence upon rechallenge (5), almonertinib demonstrates a substantially improved pulmonary safety profile, with an ILD incidence of only 0.2% as reported in the APOLLO trial (9). This stark contrast is likely rooted in fundamental differences in their chemical structures. Specifically, almonertinib’s substitution of a cyclopropyl moiety for the indole group present in osimertinib alters its metabolic processing and reduces its affinity for wild-type EGFR isoforms that are abundantly expressed in normal lung tissue (10, 11). The reduced off-target activity, a direct consequence of its distinct structure, translates into the improved pulmonary safety profile observed clinically, most notably a significantly lower risk of ILD than with osimertinib (9). Although direct comparative data are still emerging, these distinct pharmacodynamic properties establish a plausible scientific basis for the successful safety transition achieved in our patient, underscoring the importance of molecular specificity in optimizing the therapeutic index of EGFR-TKIs. Collectively, these insights offer a compelling yet hypothesis-generating explanation for the markedly different ILD risks associated with the two agents, providing a rational, mechanistic framework for the successful clinical transition observed in our patient.

The management of Osi-ILD in the adjuvant setting presents a profound therapeutic dilemma. Permanent cessation of osimertinib, mandated by safety guidelines, forfeits its unparalleled benefit in controlling the disease, as evidenced in the ADAURA trial by an 83% reduction in recurrence risk (HR 0.17) for stage II-IIIA disease (2). Adjuvant chemotherapy, the conventional alternative, yields demonstrably inferior outcomes in EGFR-mutant NSCLC. This was clearly demonstrated by the ADJUVANT trial (4), during which the 3-year disease-free survival (DFS) with vinorelbine plus cisplatin was only 36% compared to 40% with gefitinib. This efficacy gap is further magnified when compared to the landmark 5-year DFS rate of 73% achieved with adjuvant osimertinib in the ADAURA trial (2). Critically, rechallenging with osimertinib after ILD resolution poses significant risks, particularly for patients with symptomatic disease. A recent retrospective study focusing on patients with grades 2–4 Osi-ILD reported a 63% incidence of recurrent ILD upon rechallenge, with a 38% fatality rate among those who recurred (12). This alarming risk profile in severe ILD was corroborated by a Japanese real-world analysis, which observed a 37% recurrence rate specifically upon readministration to patients with grade 3–4 ILD (13). Critically, the risk extends to patients with grade 2 ILD. A separate retrospective analysis reported that even among patients with grade 2 ILD who were rechallenged with osimertinib (often with dose reduction and steroids), ILD recurred in 25% of cases (14). Consequently, osimertinib rechallenge is contraindicated in clinical practice for patients who have experienced grade 2 or higher Osi-ILD.

Faced with the high risk of osimertinib rechallenge and the suboptimal efficacy of conventional chemotherapy, our MDT unanimously endorsed switching to almonertinib as the alternative TKI therapy. This decision was grounded in two key considerations specific to this adjuvant clinical dilemma: 1) the distinct chemical structure of almonertinib (notably its cyclopropyl moiety), which is associated preclinically with an altered metabolic profile and, based on retrospective clinical safety data, correlates with a markedly lower incidence of ILD compared to osimertinib; and 2) the absence of any viable, guideline-recommended therapeutic alternative that could preserve the high efficacy of third-generation EGFR-TKI therapy in this curative setting. Although high-level evidence in the adjuvant setting remains scarce, this decision was strongly supported by compelling real-world evidence from advanced NSCLC, in which numerous studies have consistently demonstrated that sequential almonertinib after Osi-ILD achieves durable disease control and a highly favorable safety profile. For instance, in a case report and literature review of advanced NSCLC by Chen et al. (15), which described the successful use of low-dose almonertinib following grade 3 Osi-ILD in their patient and aggregated data from other published cases, the sequential treatment was associated with no ILD recurrence and a median progression-free survival of 10.2 months among the pooled cases. Similarly, a multicenter retrospective study by Wang et al. (6) also reported a favorable safety profile for sequential almonertinib after Osi-ILD in advanced NSCLC. These clinical findings align with the low ILD incidence (0.2%) noted in the APOLLO trial, further supporting a distinct safety profile attributable to almonertinib’s cyclopropyl moiety and altered metabolism (10, 11). To optimize safety, we implemented strict monitoring protocols—including confirmed radiological and symptomatic resolution of prior ILD and intensive monitoring with monthly clinical assessments and quarterly HRCT—which enabled continuous EGFR inhibition without compromising safety over 36 months of follow-up.

The therapeutic rationale for using a third-generation EGFR-TKI in this setting has been strongly bolstered by the recent presentation of the phase 3 ARTS trial (16), which demonstrated significant efficacy in the adjuvant treatment of resected stage II-IIIB EGFR-mutant NSCLC. It is important to contextualize that our clinical decision was made in early 2023, before the availability of this high-level evidence. While the ARTS trial establishes a new standard for *de novo* adjuvant therapy, our case addresses the distinct and urgent scenario of salvage therapy after life-threatening toxicity (Osi-ILD) from the previous standard-of-care agent (osimertinib). The successful outcome in this case provides early real-world evidence supporting the feasibility of this sequential strategy, which complements the evidence from prospective trials.

While this single case illustrates the potential of this sequential strategy and provides crucial early evidence, several limitations must be acknowledged to caution against premature generalization: (1) The single-case design inherently limits generalizability to broader populations and underscores the necessity of validation in larger, prospective studies. (2) The 36-month follow-up prevents assessment of long-term recurrence risks or delayed toxicities. (3) The resolution of Osi-ILD without corticosteroids may not generalize to severe cases requiring immunosuppression. (4) Echocardiography and SARS-CoV-2 RT-PCR testing were not performed at the time of ILD diagnosis, which we acknowledge as a limitation in excluding cardiac and COVID-19-related etiologies. Notwithstanding these limitations, this report offers preliminary proof-of-concept that almonertinib represents a clinically viable alternative to chemotherapy or osimertinib rechallenge in this high-risk adjuvant setting.

## Conclusion

This single case suggests that sequential almonertinib treatment after Osi-ILD can achieve sustained, long-term remission without recurrence or significant toxicity. These findings provide preliminary proof-of-concept and early real-world support for almonertinib as a viable alternative following mandatory osimertinib cessation in the adjuvant setting. However, it is imperative to emphasize that this favorable outcome, derived from a single patient, cannot be generalized. The strategy reported here represents a mechanism-driven approach to a high-stakes clinical dilemma, and its broader efficacy and safety must be rigorously evaluated in prospective clinical studies before any clinical recommendations can be made.

## Data Availability

The raw data supporting the conclusions of this article will be made available by the authors, without undue reservation.
